# The Gap Procedure: for the identification of phylogenetic clusters in HIV-1 sequence data

**DOI:** 10.1186/s12859-015-0791-x

**Published:** 2015-11-04

**Authors:** Irene Vrbik, David A. Stephens, Michel Roger, Bluma G. Brenner

**Affiliations:** 1 Department of Mathematics & Statistics, McGill University, 805 Sherbrooke Street West, Montreal, H3A 0B9 Canada; 2 Centre de recherche du CHUM, 900 rue Saint-Denis Tour Viger, H2X 0A9 Montreal, Canada; 3 Faculty of Surgery (Medicine), McGill University, 1010 Sherbrooke Street West, Montreal, H3A 2R7 Canada; 4 McGill University AIDS Centre, Jewish General Hospital, Côte-Sainte-Catherine, Montreal, H3T1E2 Canada

**Keywords:** Clustering, Phylogenetics, HIV, Genetic distance estimation

## Abstract

**Background:**

In the context of infectious disease, sequence clustering can be used to provide important insights into the dynamics of transmission. Cluster analysis is usually performed using a phylogenetic approach whereby clusters are assigned on the basis of sufficiently small genetic distances and high bootstrap support (or posterior probabilities). The computational burden involved in this phylogenetic threshold approach is a major drawback, especially when a large number of sequences are being considered. In addition, this method requires a skilled user to specify the appropriate threshold values which may vary widely depending on the application.

**Results:**

This paper presents the *Gap Procedure*, a distance-based clustering algorithm for the classification of DNA sequences sampled from individuals infected with the human immunodeficiency virus type 1 (HIV-1). Our heuristic algorithm bypasses the need for phylogenetic reconstruction, thereby supporting the quick analysis of large genetic data sets. Moreover, this fully automated procedure relies on data-driven gaps in sorted pairwise distances to infer clusters, thus no user-specified threshold values are required. The clustering results obtained by the Gap Procedure on both real and simulated data, closely agree with those found using the threshold approach, while only requiring a fraction of the time to complete the analysis.

**Conclusions:**

Apart from the dramatic gains in computational time, the Gap Procedure is highly effective in finding distinct groups of genetically similar sequences and obviates the need for subjective user-specified values. The clusters of genetically similar sequences returned by this procedure can be used to detect patterns in HIV-1 transmission and thereby aid in the prevention, treatment and containment of the disease.

**Electronic supplementary material:**

The online version of this article (doi:10.1186/s12859-015-0791-x) contains supplementary material, which is available to authorized users.

## Background

In an age overwhelmed by a massive influx of data, the need for fast and effective clustering techniques has never been greater. This endeavour is particularly important in genetics where the sheer volume of data renders many popular clustering techniques prohibitive or ineffective. The present paper aims at developing new techniques for identifying clusters of genetically similar DNA sequences and pays particular attention to HIV-infected individuals from Quebec, Canada.

In a 2013 surveillance report released by the Public Health Agency of Canada (PHAC), it is estimated that a cumulative total of 78,511 cases of HIV have been reported in Canada since 1985. Of the 2090 reported HIV cases in 2013, Quebec accounted for 21.7 %. This percentage is second only to the province of Ontario, which contributed 39.6 % of the total HIV cases in the PHAC report. Previous population-based studies involving the phylogenetic analysis of Quebec’s primary HIV infection cohort have revealed clusters that correlate with distinct social networks and risk behaviours [1–3]. In other studies performed outside of Quebec, phylogenetic clusters have been used to provide crucial insights about the spread and transmission of the disease [4–9].

Although there are a number of programs available for clustering nucleotide sequences (e.g., BLASTClust [10], UPGMA and WPGMA [11], neighbor-joining (NJ) [12], and phyclust [13]), phylogenetic approaches have been ubiquitous in the literature involving HIV-1 transmission clusters. Broadly speaking, phylogenetics is the study of evolutionary relationships among organisms or taxon [14]. There are a number of programs for inferring phylogenies including, but not limited to, PAUP* [15], BAMBE [16], BEAST [17], PHYLIP [18] RAxML [19] and MrBayes [20]. These relationships can be represented using a phylogenetic tree wherein branch lengths commonly reflect the estimated number of nucleotide substitutions between organisms. In the present study, the ‘tips’ (i.e., external nodes) of the tree represent sampled HIV-1 *pol* sequences and internal nodes can be viewed as the source of a chain of infections. We refer to all sequences rooted by a common interior node as *descendants* to the so-called *ancestor node*.

In the case of HIV, a *transmission cluster* describes a nonrandom aggregation of sequences from patients believed to share a recent common ancestor [21]. Graphically speaking, a transmission cluster corresponds to a particular branch (or monophyletic clade) in the phylogenetic tree. These transmission clusters are typically ascertained on the basis of high support—measured either by bootstrap percentages or Bayesian posterior probabilities—and sufficiently small genetic distances. One drawback of this procedure is that there is an onus on the user to determine the appropriate support/distance thresholds. In studies involving HIV, these have been reported to range anywhere from 70–99 % for bootstrap values, and 1–4.5 % for the genetic distance cutoff [21]. In addition to being data and user-specific, threshold values can also be affected by the statistical approach used to measure support. Namely, in a formal investigation conducted in [22], posterior probabilities were higher than their corresponding bootstrap values on average. Furthermore, reconstructing phylogenetic trees can be computationally intensive, especially when a large number of sequences are being considered. Despite these shortcomings, phylogenetic analysis has greatly improved our understanding of the epidemic, and remains at the forefront of cluster analysis on HIV sequences.

Herein, we present a new clustering algorithm, called the *Gap Procedure*, for identifying distinct clusters of genetically similar sequences in DNA data. This efficient and automated approach bypasses the need to estimate phylogenies and requires no user-specific threshold values. This distance-based clustering procedure relies on a dissimilarity matrix constructed using popular models for nucleotide substitution and returns a partition of the input data. Gaps in sorted pairwise distances are used to suggest groups of genetically related sequences. The frequency of these groupings are subsequently used to identify phylogenetic clusters. The efficacy and efficiency of the Gap Procedure is demonstrated on both simulated and HIV-1 *pol* sequence data.

## Methods

### Notation

Before discussing the details of our algorithm, we introduce the following notation. Let ***X***=(*X*_1_,…,*X*_*N*_) be a collection of *N* aligned sequences of length *L* where *X*_*i*_=(*x*_*i*1_,…,*x*_*iL*_). The *j*th position of the *i*th sequence, *x*_*ij*_, is recorded as an alignment gap ‘–’ or one of the International Union of Pure and Applied Chemistry (IUPAC) codes: A, C, G, T, R, Y, M, K, S, W, H, B, V, D or N [23]. Let $\mathbb {D}$ be a *N*×*N* distance matrix whose *ij*th element is equal to the genetic distance between sequence *X*_*i*_ and *X*_*j*_ denoted by *d*(*X*_*i*_,*X*_*j*_). In reference to sequence *X*_*i*_, let *d*_*i*_=(*d*(*X*_*i*_,*X*_[1]_),*d*(*X*_*i*_,*X*_[2]_),…,*d*(*X*_*i*_,*X*_[*N*−1]_)) denote the sorted vector of pairwise distances between *X*_*i*_ and *X*_*j*_ (for all *j*≠*i*) such that *d*(*X*_*i*_,*X*_[1]_)≤*d*(*X*_*i*_,*X*_[2]_)≤⋯≤*d*(*X*_*i*_,*X*_[*N*−1]_). We denote the difference between two adjacent elements in *d*_*i*_ by *δ*_*ij*_=*d*(*X*_*i*_,*X*_[*j*+1]_)−*d*(*X*_*i*_,*X*_*[j]*_). Finally, we denote a partition of the data by $\mathcal {M} = \{\mathcal {X}_{1}, \dots, \mathcal {X}_{G}\}$ where $\mathcal {X}_{g}$ is the set of sequences classified to the *g*th group.

### The algorithm

The Gap Procedure is a distance-based clustering algorithm which relies solely on a matrix of pairwise distances. There are a number of freely available packages for R [24] which can be used for evolutionary analysis. For instance, the ape package [25] contains the dist.dna() function which can compute a pairwise distance matrix for eleven substitution models; options include Jukes and Cantor 1969 (jc69) [26], Kimura 1980 (K80) [27] and Tamura and Nei 1993 (TN93) [28]. One potential drawback of this function is that it ignores sites with ambiguous nucleotides (i.e., the symbols R through N in the 15 letter IUCPAC nomenclature). Herein, distances are computed using adjusted versions of the K80 distance formula that allow fractional values for counts on the number of transitional/transversional substitutions per site. These adjusted distances—which we will refer to as aK80 distances—are described in Additional file 1.

For each individual sequence, the Gap Procedure defines a set of *nearest neighbours* that are subsequently used to determine a partition of the data. To be more specific, let *c*_*i*_= max{*δ*_*i*1_,*δ*_*i*2_,…,*δ*_*ik*_}, where *k*<*N*∗0.9. In other words, *c*_*i*_ corresponds to the largest ‘gap’ in the first 90 % of *d*_*i*_ values. As discussed in Additional file 2, this restriction was established to mitigate the effect of outlying observations. If we define *k* ^∗^ such that $\delta _{ik^{*}} \geq \delta _{\textit {ik}}\phantom {\dot {i}\!}$ for all *k*≠*k* ^∗^ and $d^{*}_{i} = d(X_{i}, X_{[k^{*}]})$, then the nearest neighbour matrix $\mathbb {N} = \{n_{\textit {ij}}\}$ can be defined as an indicator matrix whose *ij*th element is given by

(1) $$ n_{ij}=\left\{ \begin{array}{ll} 1 &\qquad \text{if}\,\, d(X_{i}, X_{j}) \leq d^{*}_{i},\\ \text{0} &\qquad \text{otherwise.} \end{array} \right.  $$

**Fig. 1:**
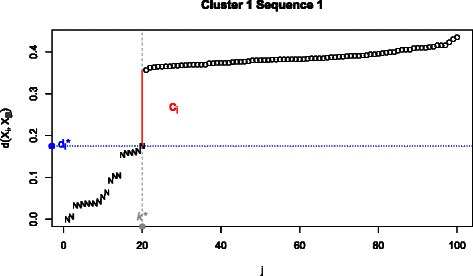
Plots the sorted pairwise distances and nearest neighbours with respect to a sequence *X*_*i*_. Plots the sorted pairwise distances with respect to the first sequence from a random run taken from Simulation 1. The vertical grey line identifies *k* ^∗^ (the position in which the largest gap is observed); the vertical red line represents *c*_*i*_ (the largest gap between sorted pairwise distances); the horizontal blue line represents $d^{*}_{i}$ (the largest pairwise distance observed before the gap). The nearest neighbours are denoted by ‘N’s



If *n*_*ij*_=1, *X*_*j*_ is said to be a neighbour to *X*_*i*_. Notice that $\mathbb {N}$ is not necessarily symmetric. A graphical representation of this definition is provided in Fig. 1. In essence, the number of times observations share a neighbour contribute to their probability of being assigned to the same group. The precise details of this partitioning procedure are codified in Algorithm 1.

### Assessing clusters

The efficacy of the Gap Procedure requires that sequences belonging to the same cluster are sufficiently similar and that the diversity between clusters is sufficiently large. In the literature, there are a variety of validation measures that can be used to test the compactness and separability of clusters, e.g., the Dunn index [29], the Calinski-Harabasz index [30], the C-index [31], the McClain-Rao index [32] and average Silhouettes [33, 34], to name a few. Herein, we assess the within-cluster distances with respect to the *g*th cluster, defined as $ S_{w}(g) = \{ d(X_{i}, X_{j}) \mid i,j \in \mathcal {X}_{g}, i <j \} $ and the corresponding between-cluster set, $ S_{b}(g) = \{ d(X_{i}, X_{j}) \mid i \in \mathcal {X}_{g}, j \notin \mathcal {X}_{g}, i < j\}. $ As a general guideline, we suggest that the Gap Procedure only be used when the 25 percentile of *S*_*b*_(*g*) is larger than the 75 percentile of *S*_*w*_(*g*) for all *g*=1,…,*G*. Graphically speaking, the side-by-side boxplots of *S*_*w*_(*g*) and *S*_*b*_(*g*) should display little to no overlap for each group found using the Gap Procedure (see Additional file 3 for examples). Future work will aim at determining if numerical validation measures, such as the indices mentioned above, can be used in place of this visual diagnostic.

### Implementation and availability

The Gap Procedure algorithm can be implemented using the GapProcedure package available on GitHub (https://github.com/vrbiki/GapProcedure). This R package includes functions for plotting the side-by-side boxplots mentioned in Section “Assessing clusters” as well as a vignette providing a step-by-step description of the algorithm and a quick demonstration. The GapProcedure package has been tested on Mac, Linux, and Windows.

## Results and discussion

In this section, we compare the results of the Gap Procedure with those obtained by the gold-standard phylogenetic threshold approach. In the threshold approach, clusters are ascertained on the basis of high clade support and low genetic distance. More precisely, given the topology of a phylogenetic tree, sequences are clustered together only if: (a) they belong to the same clade, (b) clade support (bootstrap values or posterior probabilities) exceeds *T*_*c*_, and (c) the maximum within-cluster pairwise genetic distance is below *T*_*d*_. As mentioned previously, the exact values of *T*_*c*_ and *T*_*d*_ vary between analyses. Clustering results are assessed using the Adjusted Rand Index (ARI) which measures the agreement between two partitions while accounting for chance [35]. In this particular analysis, a value of 1 corresponds to perfect agreement with the ‘true’ (i.e., simulated or expert-verified) clusters, whereas a value of 0 would be expected if clusters have been assigned at random.

Herein, phylogenetic trees were estimated using Randomized Axelerated Maximum Likelihood (RAxML) [19] and MrBayes [20]. RAxML is a program for inferring maximum likelihood trees with bootstrap support values whereas MrBayes performs a Bayesian analysis and produces summary trees with posterior probabilities. RAxML was implemented using the GTR+*Γ* model with 20 maximum likelihood searches and 100 bootstrap replicates (see RAxML manual for details). MrBayes was executed using the GTR+*I*+*Γ* substitution model and run until the average standard deviation of split frequencies—the statistic used by MrBayes to monitor convergence—droped below a value of 0.01. For notational convenience, we refer to the clusters obtained using the threshold approach on the respective trees as ‘RAxML clusters’ and ‘MrBayes clusters’. Although we used in-house code to implement the threshold approach, RAxML/MrBayes clusters could also be extracted using a program such as ClusterPicker [21]. All figures in this section were produced in R [24].

### Simulation studies

Using the seqgen() function available in the phyclust package [13], data were simulated by mutating DNA sequences along phylogenetic trees. The topology of the trees were generated at two stages *via* the ms program [13, 36]; for more details see Additional file 4. Sequences were mutated according to a General Time Reversible model which assumed rate heterogeneity and a proportion of invariable sites, i.e., the GTR+*I*+*Γ* model. For our simulation, sequences of length 800 were generated along trees made up of 4, 6, 20, or 50 transmission groups comprised of roughly 25 sequences per group^1^. For each *G*-group simulation, 100 random trees—thus 100 random data sets—were generated. Further simulations involving tree topologies different than the ones considered herein are explored in Additional file 5.

When applied to the simulated data, the Gap Procedure achieved close to perfect classification (see Table 1). Accordingly, the average ARI values were close to 1 and the average number of clusters—where “clusters” are defined to contain two or more members—roughly equalled the number of generated transmission clades (*G*). As expected, the average number of singletons, i.e., unclustered sequences, was close to 0 for all simulations. Aside from achieving excellent clustering results, the average computation time was less than a second for Simulations 1, 2, and 3, and less than 7 seconds for Simulation 4. Note that the analysis was performed using an Intel Xeon E5-1650 (3.5 GHZ) processor and includes the calculation of the pairwise distance matrix.

**Table 1:** Clustering results for the Gap Procedure on simulated data

Data			Average			
Sim	*N*	*G*	Time (in sec)	# clusters	# singletons	ARI
1	100	4	0.1108	4.25	0.04	0.9854
2	150	6	0.1370	6.39	0.04	0.9856
3	500	20	0.6073	22.49	0.13	0.9750
4	1250	50	6.6194	58.11	0.43	0.9694

The average clustering results (taken over 100 runs) obtained by the Gap Procedure when applied to the simulated data. The dissimilarity matrix was calculated using the aK80 distance formula and sequences (of length 800) were mutated according to a GTR + I + *Γ* model

**Fig. 2:**
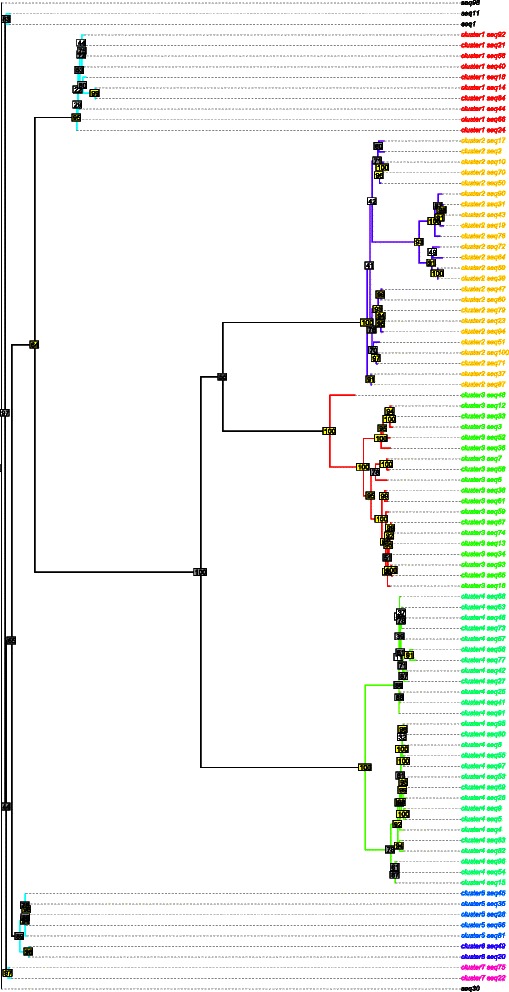
Clustering Results for RAxML with *T*_*c*_=90, *T*_*d*_=0.6. The maximum likelihood phylogenetic tree (*n*=94) produced by RAxML for Simulation 1. High, (≥90) medium (50–90) and low (<50) bootstrap values are denoted by yellow, grey and white rectangles, respectively. Cluster indices are represented by coloured tip labels; singletons are denoted in black

**Fig. 3:**
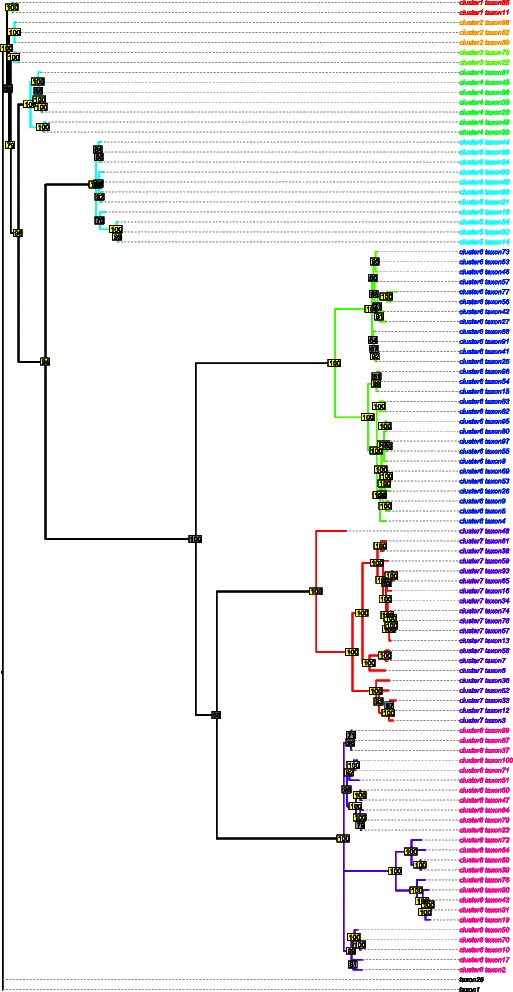
Clustering Results for MrBayes with *T*_*c*_=90, *T*_*d*_=0.6. The maximum likelihood phylogenetic tree (*n*=100) produced by RAxML for Simulation 1. High, (≥90) medium (50–90) and low (<50) posterior probabilities are denoted by yellow, grey and white rectangles, respectively. Cluster indices are represented by coloured tip labels; singletons are denoted in black

The results presented here correspond to RAxML and MrBayes clusters extracted using a clade support threshold (*T*_*c*_) of 90 % and a distance threshold (*T*_*d*_) of either 0.3 or 0.6 (the corresponding results for *T*_*d*_=0.4,0.5 are given in Additional file 6). Due to the computational complexity of these procedures, the results were based on a single run. The graphical representation of the RAxML and MrBayes clusters for Simulation 1 are shown in Figs. 2 and 3, respectively. High (≥90), medium (50–90) and low (<50) clade credibility values are denoted by yellow, grey and white rectangles, respectively. The true (i.e., simulated) transmission clusters are designated using coloured edges whereas clusters found by the threshold procedure are provided using coloured tip labels (singletons are written in black). Note that the results for RAxML contain fewer observations, since the algorithm requires that duplicated sequences be removed before analysis.

Tables 2 and 3 summarize the results obtained using the threshold approach on the phylogenetic trees estimated using RAxML and MrBayes, respectively. As the results indicate, a distance threshold value of 0.3 was inadequate to capture the simulated clusters and resulted in poor ARI values. However, when *T*_*d*_ was raised to a value of 0.6, the ARI score improved dramatically and both methods outperformed the Gap Procedure in some cases. Although potential gain in ARI values can be achieved by adopting the threshold approach, we remark that the corresponding efficacy depends greatly on the user-specified *T*_*d*_ values. Furthermore, the programs used for estimating phylogenetic trees take substantially longer to run than the Gap Procedure. For instance, the trees produced for Simulation 4 took 3 days 3 hours 26 minutes for RAxML to complete and over 15 days for MrBayes. In stark contrast, the complete analysis for the Gap Procedure took an average of 6.6 seconds. Note that the exact gain in computational time will vary depending on the data.

**Table 2:** Clustering results for RAxML on simulated data

	Sim	*T*_*c*_	*T*_*d*_	Time (in sec)	# clusters	# singletons	ARI
RAxML	1	90	0.3	2479.0	13	21	0.3662
	2	90	0.3	4654.0	13	10	0.7054
	3	90	0.3	41584.6	61	33	0.6206
	4	90	0.3	271593.7	167	70	0.4889
	1	90	0.6	2479.0	7	4	0.8757
	2	90	0.6	4654.0	9	5	0.8945
	3	90	0.6	41584.6	24	6	**0.9764**
	4	90	0.6	271593.7	54	2	**0.9922**

The clustering results (for a single run) obtained by RAxML when applied to the simulated data. The quoted run times represent the time it takes RAxML to produce a phylogenetic tree and obtain clade support values (conducted using 100 bootstrap replicates). RAxML clusters are obtained using a clade support threshold equal to *T*_*c*_ and distance thresholds of *T*_*d*_. The ARI scores in bold indicate which runs performed better than the average score obtained using the Gap Procedure

**Table 3:** Clustering results for MrBayes on simulated data

	Sim	*T*_*c*_	*T*_*d*_	Time (in sec)	# clusters	# singletons	ARI
MrBayes	1	90	0.3	3324.7	13	3	0.4642
	2	90	0.3	4243.6	19	7	0.5129
	3	90	0.3	144284.8	54	11	0.6565
	4	90	0.3	1328253.9	134	25	0.6269
	1	90	0.6	3324.7	8	2	0.8419
	2	90	0.6	4243.6	10	3	0.9011
	3	90	0.6	144284.8	24	6	**0.9768**
	4	90	0.6	1328253.9	52	3	**0.9927**

The clustering results (for a single run) obtained by MrBayes when applied to the simulated data. The quoted run times represent the time it takes MrBayes to estimate a phylogenetic tree with clade support (i.e., posterior probability) values. MrBayes clusters are obtained using a clade support threshold equal to *T*_*c*_ and distance thresholds of *T*_*d*_. The ARI scores in bold indicate which runs performed better than the average score obtained using the Gap Procedure

### Quebec HIV-1 *pol* sequence data

Multiple studies have been conducted to improve our understanding of the HIV transmission dynamics in Quebec [1–3]. Through the molecular surveillance of HIV-1 *pol* sequence data, researches were able to link high rates of onward transmission to acute/early infection. Phylogenetic analysis was performed using maximum likelihood methods *via* BioEdit [37] and MEGA2 [38]. High bootstrap values (>98 *%*) and sufficiently long branches on neighbour-joining (NJ) trees [12] were used to determine cluster membership. Manual inspection of polymorphisms and mutational motifs were used to validate clusters.

This section aims at rediscovering these transmission clusters using the automated Gap Procedure. The data, which was obtained from the provincial genotypic testing program (introduced in 1997) and the Quebec PHI cohort (established in 2001), has since been expanded to include 1517 sequences, each of length 810. Ethical approval for this cohort was granted by the Laboratoire de santé publique du Québec, and the Quebec Ministry of Health committee on confidentiality and access of information. Several subsets of this data (summarized in Table 4) were considered for the analysis. To test the efficacy of our approach, the resulting clusters were compared with those obtained by Brenner and colleagues. We henceforth refer to the latter as the ‘true’ or ‘gold-standard’ clusters.

**Table 4:** A summary of the subset data taken from the HIV-1 sequence data

				Cluster size		
Name	Description	*N*	*G* ^*b*^	1	2–4	≥5
all	Entire set	1517	169	533	108 (311)	61 (673)
men	Only males	1391	152	488	96 (276)	56 (627)
non.sing	Clustered sequences	984	169	0	108 (311)	61 (673)
nsm	Clustered males	903	152	0	96 (276)	56 (627)
big ^*a*^	Sequences clustered to big ^*a*^ clusters	673	61	0	0 (0)	61 (673)
mibc	Males clustered to big ^*a*^ clusters	627	56	0	0 (0)	56 (627)

The total number of small and large-sized clusters are listed under the headings (2–4) and (≥5). The corresponding number of sequences belonging to each heading is given in parenthesis

^a^‘big’ clusters are defined to have ≥5 members

^b^the number of clusters having ≥ 2 members

Table 5 summarizes the clustering results obtained by the Gap Procedure when applied to the six subsets of the HIV-1 data summarized in Table 4. The number of correctly and incorrectly identified singletons are given under the heading “1 $\checkmark $” and “1 ✗”, respectively. The adjacent columns provide the total number of small clusters (2–4 members) and big clusters (≥5 members); the total number of sequences belonging to the corresponding heading is reported in parentheses. Note that true singletons were removed before the ARI scores were calculated.

**Table 5:** Clustering results for the Gap Procedure on HIV-1 data

Subset	Time (in sec)	1 ✓	1 ✗	2–4	≥5	ARI
all	10.56 sec	237	16	244 (619)	61 (645)	0.9170
men	8.261 sec	225	18	215 (536)	60 (612)	0.9097
non.sing	3.086 sec	–	12	125 (351)	57 (621)	0.9325
nsm	2.470 sec	–	11	109 (303)	54 (589)	0.9320
big	0.807 sec	–	3	5 (14)	61 (656)	0.9523
mibc	0.634 sec	–	3	5 (14)	56 (610)	0.9492

The ARI scores and running times of the Gap Procedure when performed on subsets of the HIV-1 data. The number of correctly (and incorrectly) identified singletons are listed under “1$\checkmark $” (and “1✗”). The total number of for small and large-sized are listed under the headings (2–4) and (≥5). The corresponding number of sequences belonging to each class is given in parentheses

As indicated by the high ARI scores, there is a strong agreement between the true clusters and those found using our approach. In terms of cluster size, the Gap Procedure experienced some difficulty in distinguishing between small clusters and singletons. Consequently, when compared with the gold-standard, our approach found a greater number of small (2–4 member) groups. Despite this discrepancy, the Gap Procedure did well in identifying big (≥ 5 member) clusters and obtained an ARI greater than 0.9 on all data sets considered.

In addition to its excellent clustering performance, this algorithm was extremely fast when compared with the competing approaches. For example, the Gap procedure took less than a second to run on the mibc data (*N*=627,*L*=810). To produce the phylogenetic trees for the same data, RAxML and MrBayes took roughly 15 and 126 hours, respectively. In terms of clustering performance, the results of RAxML and MrBayes were highly variable (for a complete summary see Additional file 7). Using a range of threshold values, the ARIs produced by RAxML ranged anywhere from 0.0081 (with *T*_*d*_=0.01 and *T*_*c*_=99) to 0.8977 (with *T*_*d*_=0.07,0.08,0.09 or 0.1 and *T*_*c*_=90). For MrBayes clusters, the ARI ranged from as low as 0.0123 (with *T*_*d*_ = 0.01 and *T*_*c*_=98) to as high as 0.9969 (with *T*_*d*_=0.09 and *T*_*c*_=99).

## Conclusion

A distance-based clustering algorithm for genetic HIV-1 sequence data has been presented. Unlike the competing threshold approach, the Gap Procedure is fully automated (i.e., it does not require any user-specific threshold values) and relies solely on pairwise distances. Results were obtained using the GapProcedure package wherein pairwise distances are calculated using adjusted K80 distance formula. Although this is the default setting of the algorithm, alternative dissimilarity matrices may be used in its place.

When compared with RAxML and MrBayes, our algorithm showed dramatic gains in computational time, owing greatly to the fact that it bypasses the construction of a phylogenetic tree. The resulting gains in efficiency supports the quick analysis of large genetic data sets. When applied to both simulated and HIV-1 *pol* sequence data, the Gap Procedure uncovered clusters that closely agreed with true or expert-verified clusters. These encouraging results suggest that burdensome procedures involving the estimation of phylogenetic trees may not be required to infer distinct clusters of genetically similar DNA sequences.

## Endnote

^1^ Group membership is assigned according to a multinomial distribution.

## Additional files

Additional file 1
**Describes the details for computing adjusted** JC69 (aJC69)** and adjusted** K80 (aK80)** distances. Figure S1.** Side-by-side boxplots comparing K80 and aK80 distances. (PDF 228 kb)

Additional file 2
**Details regarding the Gap Procedure’s scheme for combating the effects of outliers.** **Figure S1.** Plots the sorted pairwise distances for a 3-group simulation. **Figure S2.** Plots the sorted pairwise distances for a 3-group simulation with an outlier. (PDF 109 kb)

Additional file 3
**Side-by-side boxplots.** **Figure S1.** Boxplots for the within and between cluster pairwise distances for Simulation 1. **Figure S2.** Plots the sorted pairwise distances and nearest neighbours for Simulation 1. **Figure S3.** Boxplots for the within and between cluster pairwise distances for the mibc data. **Figure S4.** Plots the sorted pairwise distances and nearest neighbours for the mibc data. (PDF 195 kb)

Additional file 4
**Describes how to construct phylogenetic trees used for generating sequence data.** **Figure S1.** Plots the step-by-step construction a 6-group phylogenetic tree. (PDF 147 kb)

Additional file 5
**Performs simulation studies involving a variety of tree topologies.** **Figure S1.** The clustering results obtained by the Gap Procedure (ARI = 0.4489) when applied to a 4-group simulation with a star-like ancestor tree. **Figure S2.** The clustering results obtained by the Gap Procedure (ARI = 0.5994) when applied to a 4-group simulation with a random bifurcating ancestor tree. **Figure S3.** The clustering results obtained by the Gap Procedure (ARI = 0.4066) when applied to a 4-group simulation with a random bifurcating ancestor tree. **Figure S4.** The clustering results obtained by the Gap Procedure (ARI = 0.6152) when applied to a 4-group simulation with a random bifurcating ancestor tree. **Table S1.** The clustering results obtained by the Gap Procedure on Simulation 1 with varying ancestor/descendant tree heights. **Table S2.** The clustering results for the Gap Procedure on Simulation 1 while relaxing the star-phylogeny assumption. (PDF 182 kb)

Additional file 6
**Cluster results for the RAxML and MrBayes clusters on simulated data with** ***T***_***d***_ **= 0.4,0.5.** **Table S1.** Cluster results for the RAxML clusters on simulated data with *T*_*d*_ = 0.4, 0.5. **Table S2.** Cluster results for the MrBayes clusters on simulated data with *T*_*d*_ = 0.4, 0.5. (PDF 114 kb)

Additional file 7
**Cluster results for the RAxML and MrBayes clusters on the** mibc **data. Table S1.** The threshold values and corresponding clustering results for RAxML on the mibc data. **Table S2.** The threshold values and corresponding clustering results for MrBayes on the mibc data. (PDF 108 kb)
