# Supplementary material for: The Gap Procedure: for the identification of phylogenetic clusters in HIV-1 sequence data
Source: BMC Bioinformatics. 2015 Nov 4;16:355. doi: 10.1186/s12859-015-0791-x (PMC4634160; doi:10.1186/s12859-015-0791-x)
Supplement: Additional file 1 — Describes the details for computing adjusted JC69 (aJC69) and adjusted K80 (aK80) distances. Figure S1. Side-by-side boxplots comparing K80 and aK80 distances. (PDF 228 kb) [file 12859_2015_791_MOESM1_ESM.pdf]

Constructing the distance matrix

Let $\boldsymbol{X} = (X_1, \ldots, X_N)$ be our data consisting of N aligned sequences of length L. Let x_{ij} be the jth site $(j = 1, \ldots, L)$ of the ith sequence $(i = 1, \ldots, N)$ such that $x_{ij} \in \mathcal{S} = \{$A, C, G, T, R, Y, M, K, S, W, H, B, V, D, N, –$\}$ (cf. IUPAC codes [3]). Many functions for computing pairwise distances between sequences ignore sites with ambiguous nucleotides, i.e., the symbols $\{$R, Y, M, K, S, W, H, B, V, D, N$\}$. Rather than deleting the incomplete information provided from these sites, we use the adjusted distance formulae described below. We first describe this adjustment under the JC69 model, followed by the more relaxed K80 model. For a nice overview of these (and other) distance formulae, see [4].

Adjusted JC69 (`aJC69`) distances

The rate matrix for the Jukes and Cantor 1969 (`JC69`) model [5] is given by:

$$\mathbb{Q}_{\texttt{JC69}} = \{q_{ij}\} = \begin{array}{c} \\ T \\ C \\ A \\ G \end{array} \begin{array}{c} \begin{array}{cccc} T & C & A & G \end{array} \\ \begin{pmatrix} \cdot & \lambda & \lambda & \lambda \\ \lambda & \cdot & \lambda & \lambda \\ \lambda & \lambda & \cdot & \lambda \\ \lambda & \lambda & \lambda & \cdot \end{pmatrix} \end{array}$$

The unadjusted distance formula is calculated using:

$$\hat{d}(X_i, X_j) = -\frac{3}{4} \log \left(1 - \frac{4}{3} \frac{p_{ij}}{L}\right) \tag{1}$$

where p_{ij} is the number of sites that are different between two sequences $X_i = (x_{i1}, x_{i2}, \ldots, x_{iN})$ and $X_j = (x_{j1}, x_{j2}, \ldots, x_{jN})$. The *adjusted* distance formula is given by:

$$\hat{\hat{d}}(X_i, X_j) = -\frac{3}{4} \log \left(1 - \frac{4}{3} \frac{\mathbb{E}\left[P_{ij} \mid X_i, X_j\right]}{L}\right) \tag{2}$$

where $\mathbb{E}\left[P_{ij} \mid X_i, X_j\right]$ is the expected number of sites that are different between the two sequences under the assumption that all nucleotides represented by the ambiguity codes are equally likely. We refer to the distances calculated using (2) as "adjusted `JC69`", or `aJC69`, distances. Let Y_s be a random variable equal to 0 if the two nucleotides are surely identical at site s, and 1, otherwise. As a simple example, suppose we have $x_{is} = R$ (base A or G) and $x_{js} = D$ (base A, G or T). The sample space and the corresponding value of Y_s are given in the table below.

Outcomes	AA	AG	AT	GA	GG	GT
P(Outcome)	$\frac{1}{6}$	$\frac{1}{6}$	$\frac{1}{6}$	$\frac{1}{6}$	$\frac{1}{6}$	$\frac{1}{6}$
Y_s	0	1	1	1	0	1

Then

$$E[Y_s \mid x_{is} = R, x_{js} = D] = 0 \cdot P(Y_s = 0 \mid x_{is} = R, x_{js} = D) + 1 \cdot P(Y_s = 1 \mid x_{is} = R, x_{js} = D)$$
$$= 0 \cdot \left(\frac{2}{6}\right) + 1 \cdot \left(\frac{4}{6}\right) = \frac{2}{3}$$

$\mathbb{E}\left[P_{ij} \mid X_i, X_j\right]$ is therefore given by

$$= \mathbb{E}\left[Y_1 \mid x_{11} = a_1, x_{21} = b_1\right] + \mathbb{E}\left[Y_2 \mid x_{12} = a_2, x_{22} = b_1\right] + \cdots + \mathbb{E}\left[Y_N \mid x_{1N} = a_N, x_{2N} = b_N\right]$$
$$= \mathbb{E}\left[Y_1 \mid a_1 b_1\right] + \mathbb{E}\left[Y_2 \mid a_2 b_1\right] + \cdots + \mathbb{E}\left[Y_N \mid a_N b_N\right] \qquad \text{(for shorthand)}$$

The conditional expected value of Y_s is given for the entire sample space in Table S1. Notice how this value is not 0 for matching ambiguous nucleotides. For instance, $\mathbb{E}\left[Y_s \mid DD\right] = 2/3$ despite the fact that the IUPAC codes are identical.

Table S1: IUPAC Nomenclature and conditional expectations of Y_s. The $\mathbb{E}\left[Y_s \mid a_s b_s\right]$ where a_s and b_s and indicated on the left and top margin, respectively. The corresponding bases for the IUPAC codes are also provided.

		b_s															
		A	C	G	T	R	M	W	S	K	Y	V	H	D	B	N	–
**Bases**	A	A				A	A	A				A	A	A		A	
	C		C				C		C		C	C	C		C	C	
	G			G		G			G	G		G		G	G	G	
	T				T			T		T	T		T	T	T	T	
	A	0	1	1	1	$\frac{1}{2}$	$\frac{1}{2}$	$\frac{1}{2}$	1	1	1	$\frac{1}{3}$	$\frac{1}{3}$	$\frac{1}{3}$	1	$\frac{3}{4}$	1
	C	1	0	1	1	1	$\frac{1}{2}$	1	$\frac{1}{2}$	1	$\frac{1}{2}$	$\frac{1}{3}$	$\frac{1}{3}$	1	$\frac{1}{3}$	$\frac{3}{4}$	1
	G	1	1	0	1	$\frac{1}{2}$	$\frac{1}{2}$	$\frac{1}{2}$	1	1	1	$\frac{1}{3}$	1	$\frac{1}{3}$	$\frac{1}{3}$	$\frac{3}{4}$	1
	T	1	1	1	0	$\frac{1}{2}$	$\frac{1}{2}$	$\frac{1}{2}$	1	1	1	1	$\frac{1}{3}$	$\frac{1}{3}$	$\frac{1}{3}$	$\frac{3}{4}$	1
a_s	R	$\frac{1}{2}$	1	$\frac{1}{2}$	1	$\frac{1}{2}$	$\frac{3}{4}$	$\frac{3}{4}$	$\frac{3}{4}$	$\frac{3}{4}$	1	$\frac{2}{3}$	$\frac{5}{6}$	$\frac{2}{3}$	$\frac{5}{6}$	$\frac{6}{8}$	1
	M	$\frac{1}{2}$	$\frac{1}{2}$	1	1	$\frac{3}{4}$	$\frac{1}{2}$	$\frac{3}{4}$	$\frac{3}{4}$	1	$\frac{3}{4}$	$\frac{2}{3}$	$\frac{2}{3}$	$\frac{5}{6}$	$\frac{5}{6}$	$\frac{6}{8}$	1
	W	$\frac{1}{2}$	1	1	$\frac{1}{2}$	$\frac{3}{4}$	$\frac{3}{4}$	$\frac{1}{2}$	1	$\frac{3}{4}$	$\frac{3}{4}$	$\frac{5}{6}$	$\frac{2}{3}$	$\frac{2}{3}$	$\frac{5}{6}$	$\frac{6}{8}$	1
	S	1	$\frac{1}{2}$	$\frac{1}{2}$	1	$\frac{3}{4}$	$\frac{3}{4}$	1	$\frac{1}{2}$	$\frac{3}{4}$	$\frac{3}{4}$	$\frac{2}{3}$	$\frac{5}{6}$	$\frac{5}{6}$	$\frac{2}{3}$	$\frac{6}{8}$	1
	K	1	1	$\frac{1}{2}$	$\frac{1}{2}$	$\frac{3}{4}$	1	$\frac{3}{4}$	$\frac{3}{4}$	$\frac{1}{2}$	$\frac{3}{4}$	$\frac{5}{6}$	$\frac{5}{6}$	$\frac{2}{3}$	$\frac{2}{3}$	$\frac{6}{8}$	1
	Y	1	$\frac{1}{2}$	1	$\frac{1}{2}$	1	$\frac{3}{4}$	$\frac{3}{4}$	$\frac{3}{4}$	$\frac{3}{4}$	$\frac{1}{2}$	$\frac{5}{6}$	$\frac{2}{3}$	$\frac{5}{6}$	$\frac{2}{3}$	$\frac{6}{8}$	1
	V	$\frac{2}{3}$	$\frac{2}{3}$	$\frac{2}{3}$	1	$\frac{2}{3}$	$\frac{2}{3}$	$\frac{5}{6}$	$\frac{2}{3}$	$\frac{5}{6}$	$\frac{5}{6}$	$\frac{2}{3}$	$\frac{7}{9}$	$\frac{7}{9}$	$\frac{7}{9}$	$\frac{3}{4}$	1
	H	$\frac{2}{3}$	$\frac{2}{3}$	1	$\frac{2}{3}$	$\frac{5}{6}$	$\frac{2}{3}$	$\frac{2}{3}$	$\frac{5}{6}$	$\frac{5}{6}$	$\frac{2}{3}$	$\frac{7}{9}$	$\frac{2}{3}$	$\frac{7}{9}$	$\frac{7}{9}$	$\frac{3}{4}$	1
	D	$\frac{2}{3}$	1	$\frac{2}{3}$	$\frac{2}{3}$	$\frac{2}{3}$	$\frac{5}{6}$	$\frac{2}{3}$	$\frac{5}{6}$	$\frac{2}{3}$	$\frac{5}{6}$	$\frac{7}{9}$	$\frac{7}{9}$	$\frac{2}{3}$	$\frac{7}{9}$	$\frac{3}{4}$	1
	B	1	$\frac{2}{3}$	$\frac{2}{3}$	$\frac{2}{3}$	$\frac{5}{6}$	$\frac{5}{6}$	$\frac{5}{6}$	$\frac{2}{3}$	$\frac{2}{3}$	$\frac{2}{3}$	$\frac{7}{9}$	$\frac{7}{9}$	$\frac{7}{9}$	$\frac{2}{3}$	$\frac{3}{4}$	1
	N	$\frac{3}{4}$	$\frac{3}{4}$	$\frac{3}{4}$	$\frac{3}{4}$	$\frac{3}{4}$	$\frac{3}{4}$	$\frac{3}{4}$	$\frac{3}{4}$	$\frac{3}{4}$	$\frac{3}{4}$	$\frac{3}{4}$	$\frac{3}{4}$	$\frac{3}{4}$	$\frac{3}{4}$	$\frac{3}{4}$	1
	–	1	1	1	1	1	1	1	1	1	1	1	1	1	1	1	0

Adjusted K80 (aK80) distances

The rate matrix for the Kimura 1980 (K80) model [6] is given by:

$$Q_{\texttt{K80}} = \{q_{ij}\} = \begin{array}{c} \\ T \\ C \\ A \\ G \end{array} \begin{array}{c} \begin{array}{cccc} T & C & A & G \end{array} \\ \begin{pmatrix} \cdot & \alpha & \beta & \beta \\ \alpha & \cdot & \beta & \beta \\ \beta & \beta & \cdot & \alpha \\ \beta & \beta & \alpha & \cdot \end{pmatrix} \end{array}$$

The adjusted K80, or aK80, distances are calculated by:

$$\hat{d}(X_i, X_j) = -\frac{1}{2}\log\left(1 - 2\frac{\mathbb{E}[S]}{L} - \frac{\mathbb{E}[V]}{L}\right) - \frac{1}{4}\log\left(1 - 2\frac{\mathbb{E}[V]}{L}\right)$$

where

$$\mathbb{E}[S] = \text{the expected number of sites with transitional differences}$$
$$\mathbb{E}[V] = \text{the expected number of sites with two transversional differences}$$

Let W_s be a random variable equal to 1 if the two nucleotides at site s are surely a transition (i.e. A $\leftrightarrow$ G, C $\leftrightarrow$ T) and 0 otherwise. Similarly, we define Z_s to be a random variable equal to 1 if the two nucleotides at site s are surely a transversion (i.e. A $\leftrightarrow$ T/C, G $\leftrightarrow$ T/C, C $\leftrightarrow$ A/G, T $\leftrightarrow$ A/G) and 0 otherwise. Again, suppose $x_{is} = R$ and $x_{js} = D$. The sample space and the corresponding values of W_s and Z_s are given in the table below.

Outcomes	AA	AG	AT	GA	GG	GT
P(Outcome)	$\frac{1}{6}$	$\frac{1}{6}$	$\frac{1}{6}$	$\frac{1}{6}$	$\frac{1}{6}$	$\frac{1}{6}$
W_s	0	1	0	1	0	0
Z_s	0	0	1	0	0	1

For example, $E[W_s \mid x_{is} = R, x_{js} = D]$ is given by

$$= 0 \cdot P(W_s = 0 \mid x_{is} = R, x_{js} = D) + 1 \cdot P(W_s = 1 \mid x_{is} = R, x_{js} = D)$$
$$= 0 \cdot \left(\frac{4}{6}\right) + 1 \cdot \left(\frac{2}{6}\right) = \frac{1}{3}$$

and $E[Z_s \mid x_{is} = R, x_{js} = D]$ is given by

$$= 0 \cdot P(Z_s = 0 \mid x_{is} = R, x_{js} = D) + 1 \cdot P(Z_s = 1 \mid x_{is} = R, x_{js} = D)$$
$$= 0 \cdot \left(\frac{4}{6}\right) + 1 \cdot \left(\frac{2}{6}\right) = \frac{1}{3}$$

The following expectations are therefore given by

$$\mathbb{E}[S \mid X_i X_j] = \mathbb{E}[W_1 \mid a_1 b_1] + \mathbb{E}[W_2 \mid a_2 b_1] + \cdots + \mathbb{E}[W_N \mid a_N b_N]$$
$$\mathbb{E}[V \mid X_i X_j] = \mathbb{E}[Z_1 \mid a_1 b_1] + \mathbb{E}[Z_2 \mid a_2 b_1] + \cdots + \mathbb{E}[Z_N \mid a_N b_N]$$

Figure S1 plots the aK80 and K80 pairwise distances between sequences from the mibc data set. As evident from these plots, a larger genetic diversity can be discovered when ambiguous sites are taken into account.

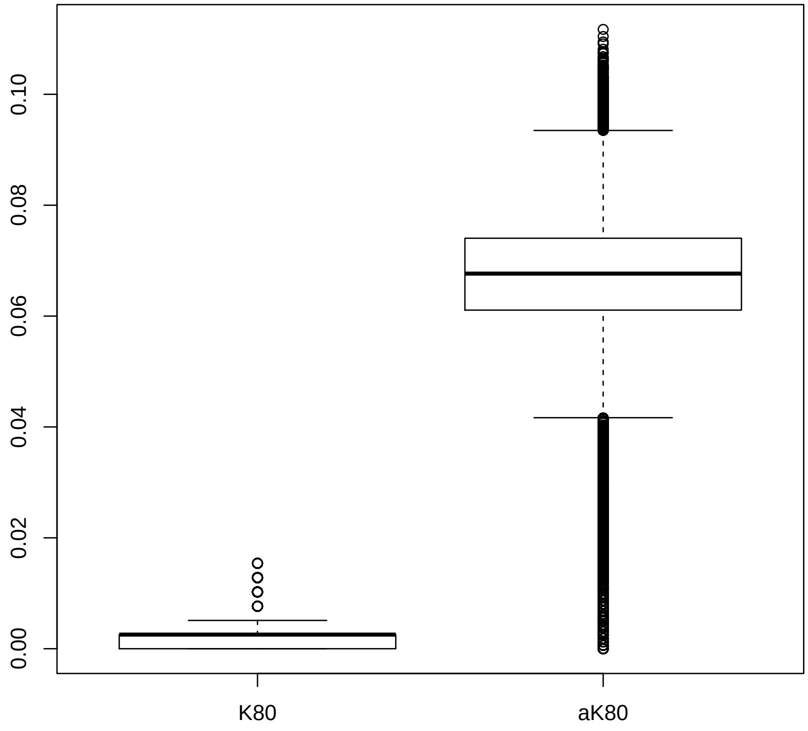

Figure S1: Boxplots of pairwise distances. Boxplots of the pairwise distances between sequences from the mibc data set (containing 627 sequences of length 810). The boxplot on the left uses the `K80` pairwise distances computed using the `dist.dna()` function from the ape package. The boxplot on the right uses the `aK80` distances described herein.

References

[1] Dondoshansky, I., Wolf, Y.: BLASTclust. Bioinformatics Toolkit, Max-Planck Institute for Developmental Biology: http://toolkit.tuebingen.mpg.de/blastclust (2008–2015)

[2] Swofford, D.L.: PAUP*. Phylogenetic Analysis Using Parsimony (and other methods). version 4. (2003)

[3] Cornish-Bowden, A.: IUPAC-IUB symbols for nucleotide nomenclature. Nucleic Acids Res **13**, 3021–3030 (1985)

[4] Yang, Z.: Computational Molecular Evolution. Oxford University Press Oxford, Oxford (2006)

[5] Jukes, T.H., Cantor, C.R.: Evolution of protein molecules. Mammalian Protein Metabolism, 21–132 (1969)

[6] Kimura, M.: A simple method for estimating evolutionary rates of base substitutions through comparative studies of nucleotide sequences. Journal of molecular evolution **16**(2), 111–120 (1980)
