# Supplementary material for: The Gap Procedure: for the identification of phylogenetic clusters in HIV-1 sequence data
Source: BMC Bioinformatics. 2015 Nov 4;16:355. doi: 10.1186/s12859-015-0791-x (PMC4634160; doi:10.1186/s12859-015-0791-x)
Supplement: Additional file 2 — Details regarding the Gap Procedure’s scheme for combating the effects of outliers. Figure S1. Plots the sorted pairwise distances for a 3-group simulation. Figure S2. Plots the sorted pairwise distances for a 3-group simulation with an outlier. (PDF 109 kb) [file 12859_2015_791_MOESM2_ESM.pdf]

An adjustment for outliers

Figure S1 gives a graphical representation of a typical 'gap' present in sorted pairwise distances for a 3-group simulation. Specifically, the left panel in Figure S1 corresponds to the $\boldsymbol{d}_{(i\cdot)}$ vector for a sequence, X_i, belonging to the group labelled using green circles (○). The vertical grey line indicates the boundary determined by the largest gap and hence marks the boundary for X_i's nearest neighbours. Similar plots for reference sequences belonging to the black (△) and red (+) group are given in the middle and right panels of Figure S1, respectively. Note that this gap performs well at separating members from non-members; however, gaps are not necessarily present in the sorted distances between the groups of non-members. For example, the points lying to the right of the grey line in the left panel of Figure S1 intermix the distances corresponding to the red and black group.

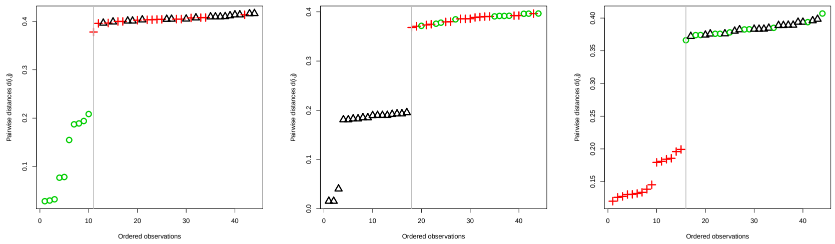

Figure S1: Plots of sorted pairwise distances for a 3-group simulation. Plots the values of $\boldsymbol{d}_{i\cdot}$ for reference sequences belonging to the green (left), black (middle) and red (right) group.

For our implementation of the Gap Procedure, only the first 90% of sorted distances are considered during the gap search. This adjustment was implemented to ensure that large gaps produced by outlying observations did not divert attention from other meaningful gaps between clusters. Suppose, for example, an outlier was added to the three group simulation depicted in Figure S1. As shown in Figure S2, the inclusion of this outlier results in the remaining observations being labeled as neighbours to the reference sequence. To combat this, we disregard large gaps falling in the upper portion of the plotted distances, and focus on gaps falling in the lower 90% of sorted data. Once this alteration has been made, the algorithm can properly identify the natural jump between black and non-black cluster members (cf. Figure S2).

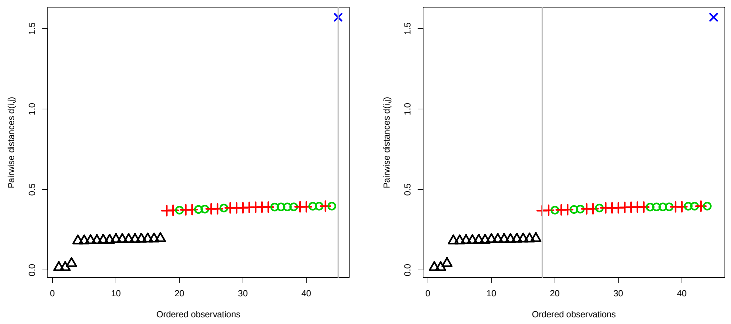

Figure S2: Plots of sorted pairwise distances for a 3-group simulation with an outlier. Left: The vertical line indicates the place where the largest gap between distances occurs. Right: The vertical line marks the largest gap in the first 90% of sorted distances.
