# Supplementary material for: The Gap Procedure: for the identification of phylogenetic clusters in HIV-1 sequence data
Source: BMC Bioinformatics. 2015 Nov 4;16:355. doi: 10.1186/s12859-015-0791-x (PMC4634160; doi:10.1186/s12859-015-0791-x)
Supplement: Additional file 3 — Side-by-side boxplots. Figure S1. Boxplots for the within and between cluster pairwise distances for Simulation 1. Figure S2. Plots the sorted pairwise distances and nearest neighbours for Simulation 1. Figure S3. Boxplots for the within and between cluster pairwise distances for the mibc data. Figure S4. Plots the sorted pairwise distances and nearest neighbours for the mibc data. (PDF 195 kb) [file 12859_2015_791_MOESM3_ESM.pdf]

Side-by-side boxplots

The following plots were produced using the `boxplotWB()` and `GapPlot()` function available in the GapProcedure package. This package is freely available on GitHub https://github.com/vrbiki/GapProcedure.

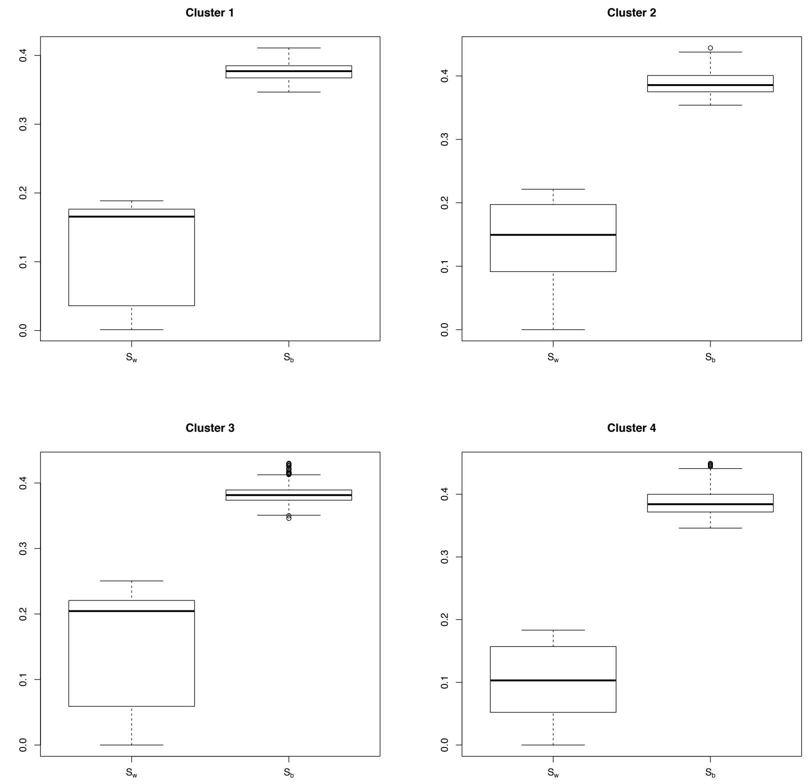

Figure S1: Boxplots for the within and between cluster pairwise distances for Simulation 1. Plots the side-by-side boxplots of the within-cluster distances, $S_w(g)$, and between-cluster distances, $S_b(g)$ $(g = 1, 2, 3, 4)$, for a four-group simulation with a star-phylogeny ancestor tree and r_{AD} =0.8.

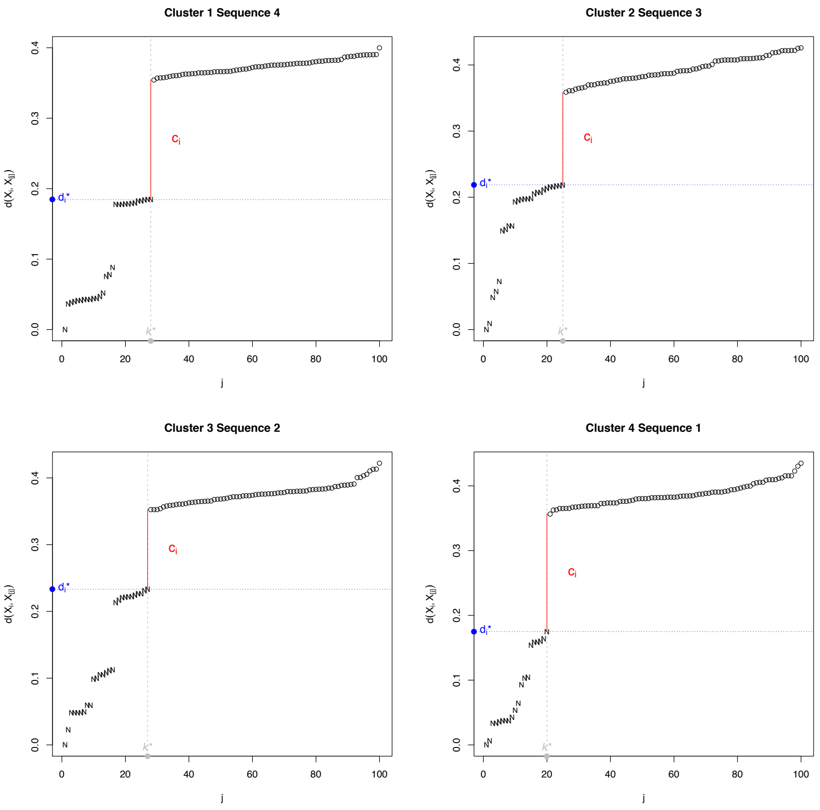

Figure S2: Plots the sorted pairwise distances and nearest neighbours for Simulation 1. Plots the sorted pairwise distances with respect to the sequence and cluster indicated in the plot title. The vertical grey line indicates the position in which the largest gap is observed; the vertical red line represents c_i, the largest gap between sorted pairwise distances; the horizontal blue line represents d_i^* the largest pairwise distance observed before the gap. The nearest neighbours are denoted by 'N's.

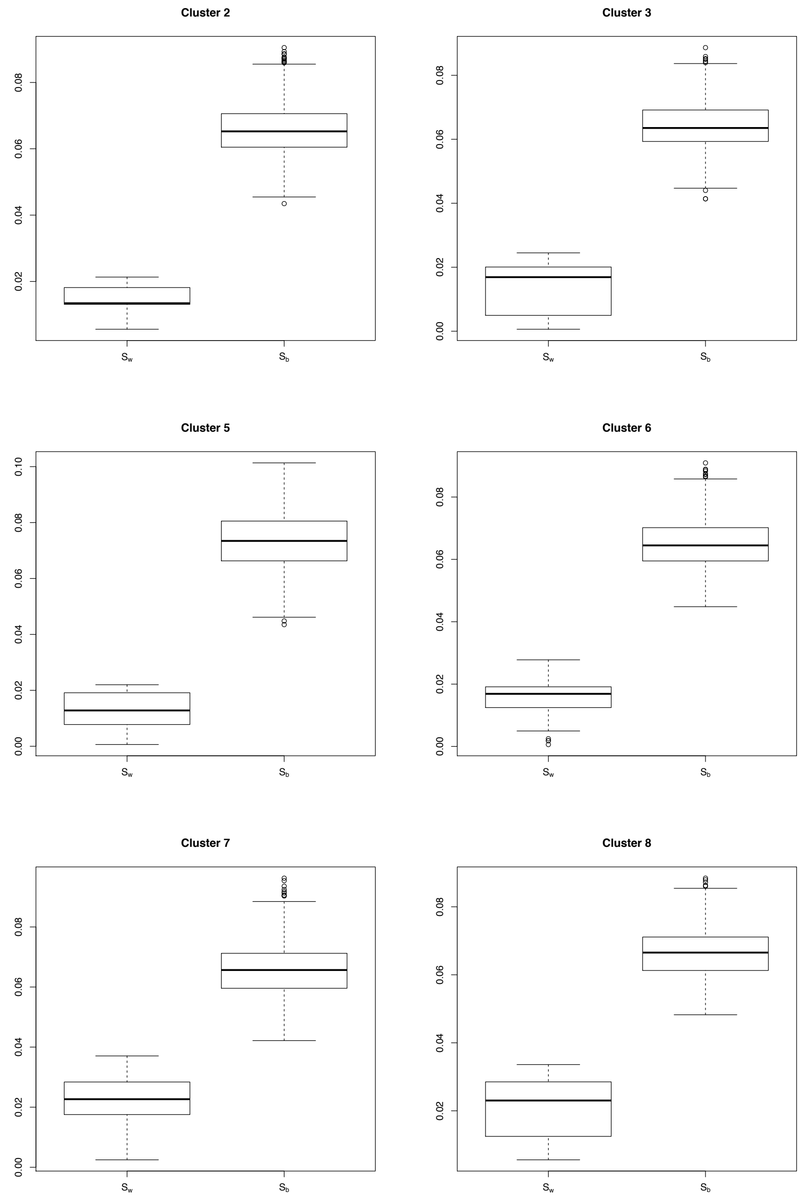

Figure S3: Boxplots for the within and between cluster pairwise distances for the mibc data. Plots the side-by-side boxplots of the within-cluster distances, $S_w(g)$ and between-cluster distances, $S_b(g)$, for six big clusters ($g = 2, 3, 5, 6, 7, 8$) found by the Gap Procedure on the mibc data.

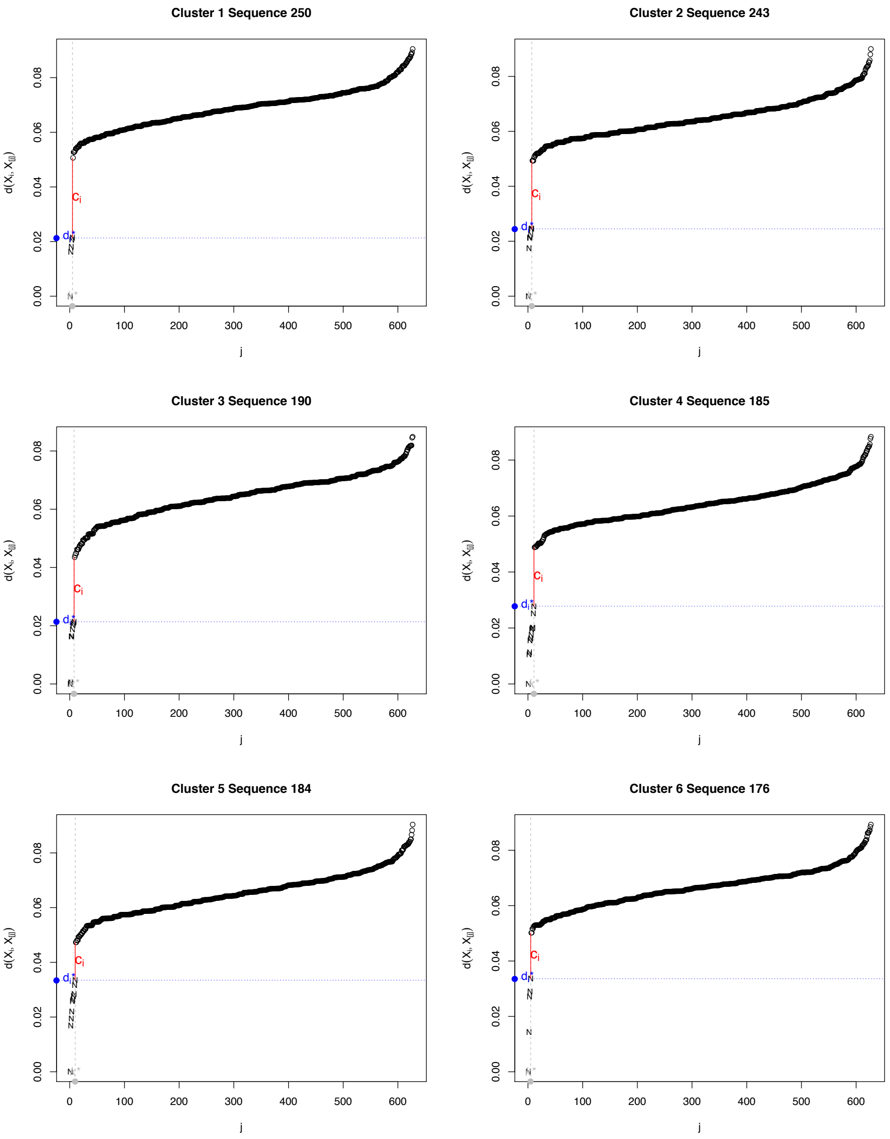

Figure S4: Plots the sorted pairwise distances and nearest neighbours for the mibc data. Plots the sorted pairwise distances with respect to the sequence and cluster indicated in the plot title. The vertical grey line indicates the position in which the largest gap is observed; the vertical red line represents c_i, the largest gap between sorted pairwise distances; the horizontal blue line represents d_i^* the largest pairwise distance observed before the gap. The nearest neighbours are denoted by ‘N’s.
