# Supplementary material for: The Gap Procedure: for the identification of phylogenetic clusters in HIV-1 sequence data
Source: BMC Bioinformatics. 2015 Nov 4;16:355. doi: 10.1186/s12859-015-0791-x (PMC4634160; doi:10.1186/s12859-015-0791-x)
Supplement: Additional file 4 — Describes how to construct phylogenetic trees used for generating sequence data. Figure S1. Plots the step-by-step construction a 6-group phylogenetic tree. (PDF 147 kb) [file 12859_2015_791_MOESM4_ESM.pdf]

Simulating sequences and phylogenetic trees

Using the `seqgen()` function available in the `phyclust` package [1], data were simulated by mutating DNA sequences along phylogenetic trees. The topology of the trees were generated at two stages *via* the `ms` program [1, 2]. At the first stage we randomly generated a root sequence and a star-like *ancestor tree* having equal branch lengths and tips equal to the number of desired clusters. The tip label $\mathtt{a_g}$ denotes the ancestor node, or recent common ancestor, of the gth cluster. The second stage generates a *descendant tree* for each cluster which is rooted at $\mathtt{a_g}$. The number of tips in the gth descendant tree represents the number of members in cluster g. The *complete tree* is obtained by attaching the G descendant trees to the tips of the ancestor tree. For instance, Figure S1 shows the complete tree (right panel) created by binding six randomly generating descendent trees (example in middle panel) to the corresponding tips on the ancestor tree (left panel).

Standard phylogenetic trees typically involve bifurcating branches, i.e., exactly two diverging sequences per node. It may be the case, however, that the rapid diversification of HIV is better modeled using multifurcating trees [3]. For this reason, our simulations were created using star-like ancestor trees which take on a "pitchfork" structure (cf. Figure S1). In our context, this topology indicates that patients have simultaneously (or very rapidly) diverged from a common root [4]. The height of the ancestor tree, denoted by r_{A}, determines the diversity between the ancestor node sequences, while the height of the descendant trees, r_{D}, determines the diversity between sequences belonging to the same group. The proportion of the ancestor to descendent height is determined by the growth rate ratio $r_{\mathrm{AD}} = r_{\mathrm{A}}/(r_{\mathrm{A}} + r_{\mathrm{D}})$. Herein, complete trees are scaled to have total height one; consequently, $r_{\mathrm{AD}} = r_{\mathrm{A}}$.

Based on the topology of the tree, we generated DNA sequences with `seqgen()`. Sequences were mutated according to a GTR model which assumes that sites evolve according to random variable $R \sim \Gamma(\alpha, \beta)$ (parameterized such that $E[R] = \alpha/\beta$). To avoid too many parameters, it is commonplace to assume a Gamma distribution parameterized by a single shape parameter α, where $\alpha = \beta$. In addition, a proportion of sites (p) are assumed to be invariable. Together, these specifications are referred to as the GTR + I + Γ model. In accordance with [5] we set these parameters to values typical for HIV-1 Subtype B on the *pol* gene; namely, we set $\alpha = 0.7589$, the proportion of invariant sites to $p = 0.4817$ and specify the rate matrix to:

$$\mathbb{Q}_{GTR} = \begin{array}{c} \\ T \\ C \\ A \\ G \end{array} \begin{array}{c} \begin{array}{cccc} T & C & A & G \end{array} \\ \begin{pmatrix} \cdot & 14.50 & 1.44 & 1.00 \\ 14.50 & \cdot & 3.37 & 1.21 \\ 1.44 & 3.37 & \cdot & 14.50 \\ 1.00 & 1.21 & 14.50 & \cdot \end{pmatrix} \end{array} \begin{array}{c} \begin{array}{cccc} T & C & A & G \end{array} \\ \begin{pmatrix} 0.22 & 0 & 0 & 0 \\ 0 & 0.17 & 0 & 0 \\ 0 & 0 & 0.39 & 0 \\ 0 & 0 & 0 & 0.22 \end{pmatrix} \end{array}$$

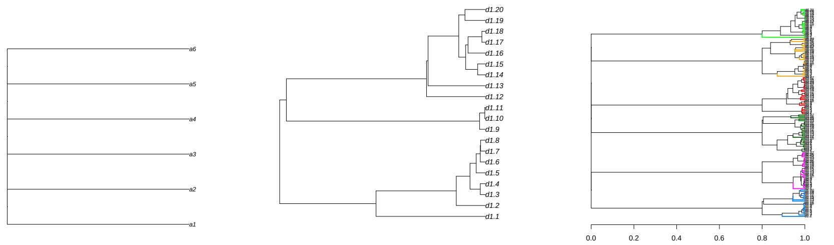

Figure S1: Constructing a 6-group phylogenetic tree. The complete tree (right) is constructed by attaching six randomly generated descendent trees (e.g., middle) to the tips of the corresponding tips of the ancestor tree (left).

References

[1] Chen, W.C.: Overlapping Codon Model, Phylogenetic Clustering, and Alternative Partial Expectation Conditional Maximization Algorithm. (2011). http://gradworks.umi.com/34/73/3473002.html

[2] Hudson, R.R.: Generating samples under a Wright–Fisher neutral model of genetic variation. Bioinformatics **18**(2), 337–338 (2002)

[3] Salemi, M., Vandamme, A.-M.: The Phylogenetic Handbook: a Practical Approach to DNA and Protein Phylogeny. Cambridge University Press, Cambridge (2003)

[4] Page, R.D., Holmes, E.C.: Molecular Evolution: a Phylogenetic Approach. Blackwell Science, Oxford (1998)

[5] Posada, D., Crandall, K.A.: Selecting models of nucleotide substitution: an application to human immunodeficiency virus 1 (HIV-1). Molecular Biology and Evolution **18**(6), 897–906 (2001)
