# Supplementary material for: The Gap Procedure: for the identification of phylogenetic clusters in HIV-1 sequence data
Source: BMC Bioinformatics. 2015 Nov 4;16:355. doi: 10.1186/s12859-015-0791-x (PMC4634160; doi:10.1186/s12859-015-0791-x)
Supplement: Additional file 5 — Performs simulation studies involving a variety of tree topologies. Figure S1. The clustering results obtained by the Gap Procedure (ARI = 0.4489) when applied to a 4-group simulation with a star-like ancestor tree. Figure S2. The clustering results obtained by the Gap Procedure (ARI = 0.5994) when applied to a 4-group simulation with a random bifurcating ancestor tree. Figure S3. The clustering results obtained by the Gap Procedure (ARI = 0.4066) when applied to a 4-group simulation with a random bifurcating ancestor tree. Figure S4. The clustering results obtained by the Gap Procedure (ARI = 0.6152) when applied to a 4-group simulation with a random bifurcating ancestor tree. Table S1. The clustering results obtained by the Gap Procedure on Simulation 1 with varying ancestor/descendant tree heights. Table S2. The clustering results for the Gap Procedure on Simulation 1 while relaxing the star-phylogeny assumption. (PDF 182 kb) [file 12859_2015_791_MOESM5_ESM.pdf]

Simulations with varying tree topologies

In the simulation studies, phylogenetic trees (which are scaled to have a height of 1) are constructed by attaching randomly generated descendant trees (having a height r_{D}) to the tips of a star-shaped ancestor tree (having a height r_{A}). Here we investigate the affect that different tree topologies have on the performance of the Gap Procedure.

Adjusting the relative height of the ancestor/descendant trees

As demonstrated in Table S1, higher ARI values are observed as the similarity (resp. diversity) among sequences within (resp. between) clusters grow. To put another way, the value of r_{AD} needs to be large enough (approximately larger than 0.7) in order for the Gap Procedure to agree closely with the simulated clusters. Figure S1 plots a randomly generated complete tree with $r_{\mathrm{AD}} = 0.3$; simulated clusters are denoted by tip labels (e.g., Cluster2.s corresponds to the sth sequence in simulated cluster 2) and tip numbers/colours correspond to cluster labels found using the Gap Procedure. In this simulation, small ARI values were typically a result of the Gap Procedure finding "lower-level" clusters. For instance, the simulated cluster associated with ancestor node a4 is split into two separate clusters (Cluster 4 and 13) and one singleton. Although the this partition corresponds to a fairly low ARI value (0.4489), when compared to the true clusters one could argue that the Gap Procedure is discovering reasonable subgroups within the simulated clusters.

Relaxing the star-phylogeny assumption

This section investigates the efficacy of the Gap Procedure when the pitchfork structure imposed on the ancestor tree is relaxed. When the ancestor tree is generated in a fashion similar to the descendant trees, we find that Gap Procedure attains lower ARI scores on average (see Table S2). These poor results are a byproduct of the Gap Procedure finding clusters corresponding to clades located higher (closer to the root) and/or lower (closer to the tips) on the phylogenetic tree. For instance, Figure S2 and S3 displays the cases when the number of groups is over-estimated and under-estimated, respectively. Although the ARI values are small (0.5994 and 0.4066, respectively), we reiterate that this touches upon the larger issue of what clades correspond to the "best" partition of a tree. For example, one could argue that the clusters found by the Gap Procedure in Figure S4 define a more natural partition of the data since there is very little diversification between ancestor nodes a3 and a4. Conversely, simulated cluster 2 exhibits signs of sub-clustering which the Gap Procedure identifies as two separate groups.

Figure S1: The clustering results obtained by the Gap Procedure when applied to a 4-group simulation with a star-like ancestor tree having height $r_{\mathrm{A}} = 0.3$ and random descendant trees having height $r_{\mathrm{D}} = 0.7$. The true (i.e., simulated) cluster memberships are given in the tip labels (e.g., Cluster2.s corresponds to a sequence in cluster 2) and tip colours correspond to the clusters found using the Gap Procedure (the cluster label is indicated within the circle frame). The corresponding ARI value is 0.4489.

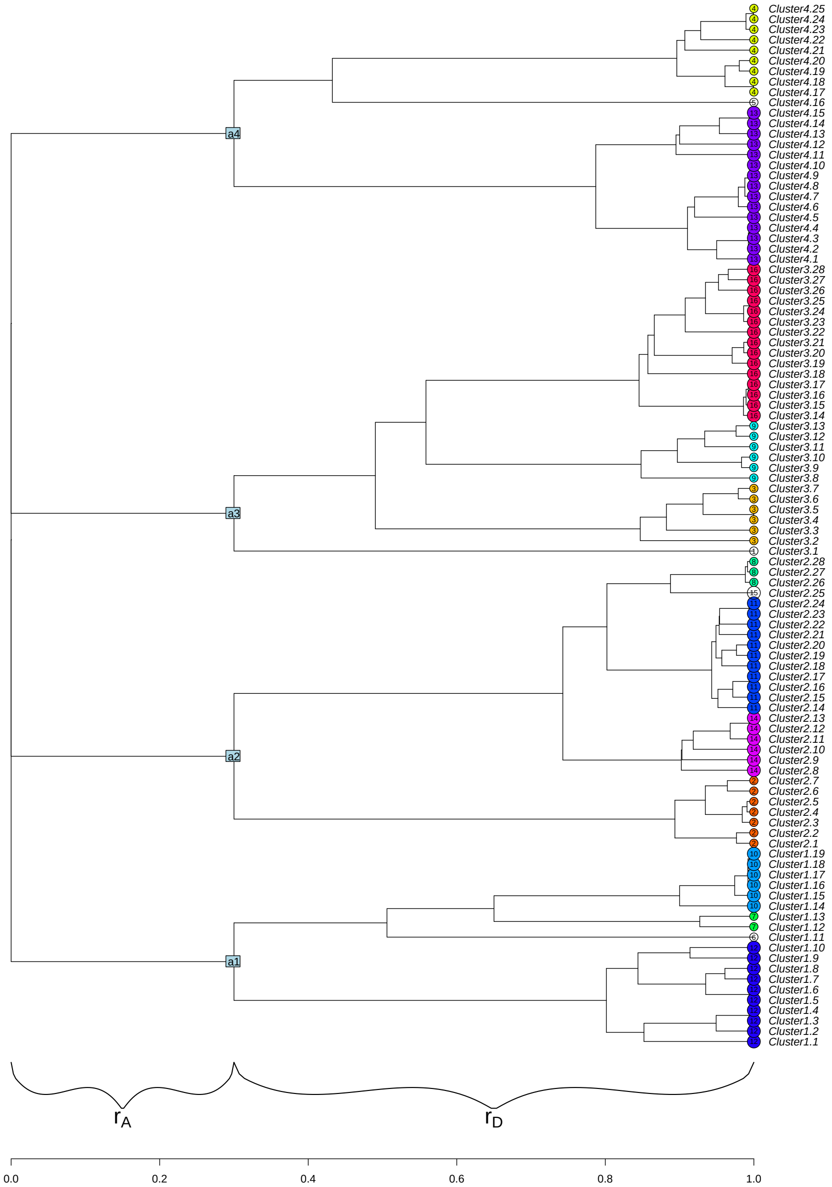

Figure S2: The clustering results obtained by the Gap Procedure when applied to a 4-group simulation with a random bifurcating ancestor tree having height $r_{\mathrm{A}} = 0.8$ and random descendant trees having height $r_{\mathrm{D}} = 0.2$. The true (i.e., simulated) cluster memberships are given in the tip labels (e.g., Cluster2.s corresponds to a sequence in cluster 2) and tip colours correspond to the clusters found using the Gap Procedure (the cluster label is indicated within the circle frame). The corresponding ARI value is 0.5994.

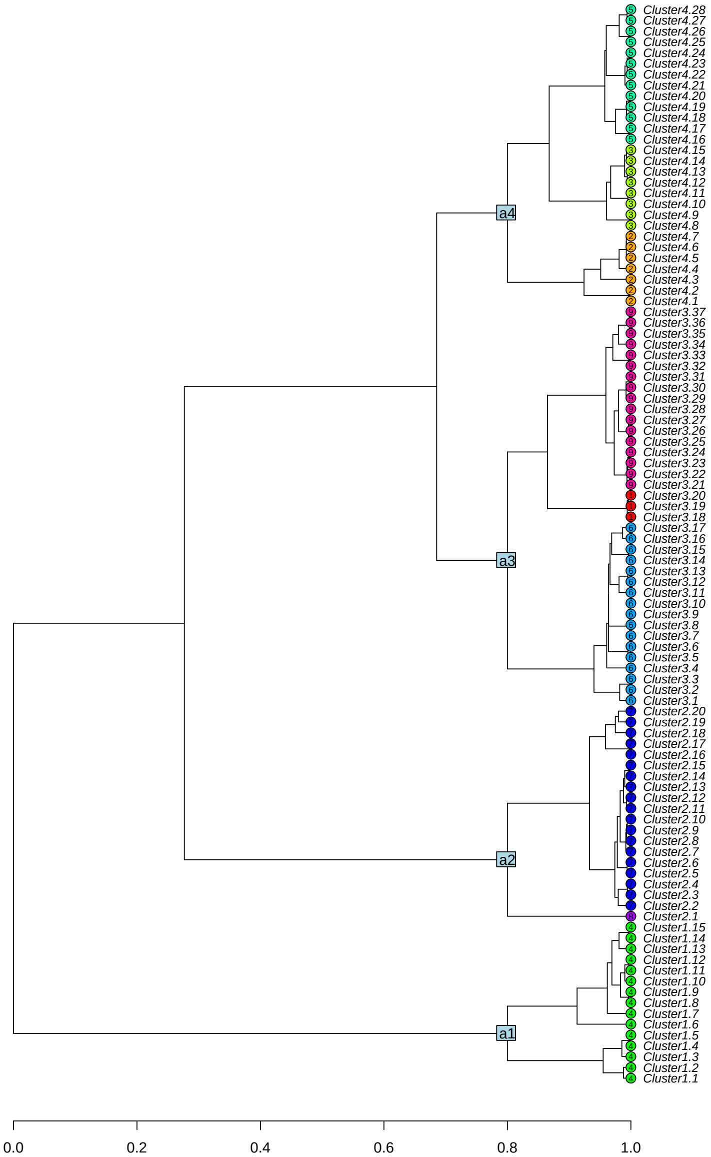

Figure S3: The clustering results obtained by the Gap Procedure when applied to a 4-group simulation with a random bifurcating ancestor tree having height $r_{\mathrm{A}} = 0.8$ and random descendant trees having height $r_{\mathrm{D}} = 0.2$. The true (i.e., simulated) cluster memberships are given in the tip labels (e.g., Cluster2.s corresponds to a sequence in cluster 2) and tip colours correspond to the clusters found using the Gap Procedure (the cluster label is indicated within the circle frame). The corresponding ARI value is 0.4066.

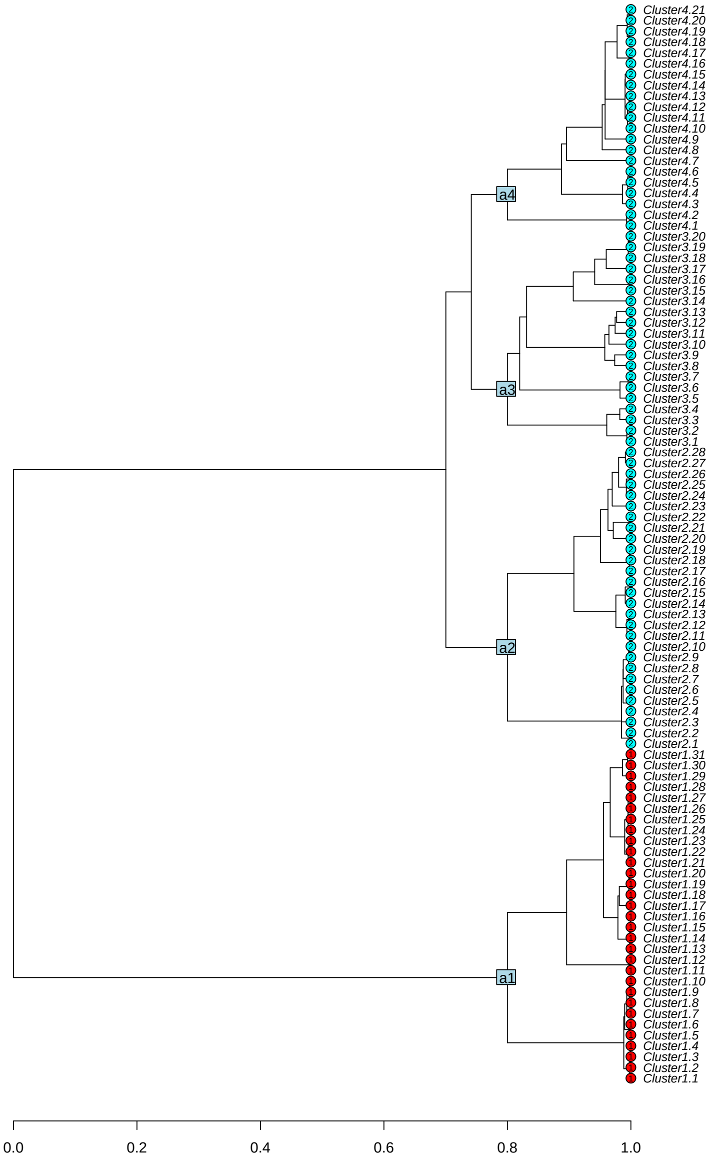

Figure S4: The clustering results obtained by the Gap Procedure when applied to a 4-group simulation with a random bifurcating ancestor tree having height $r_{\mathrm{A}} = 0.8$ and random descendant trees having height $r_{\mathrm{D}} = 0.2$. The true (i.e., simulated) cluster memberships are given in the tip labels (e.g., Cluster2.s corresponds to a sequence in cluster 2) and tip colours correspond to the clusters found using the Gap Procedure (the cluster label is indicated within the circle frame). The corresponding ARI value is 0.6152. The internal nodes `a1`, `a2`, ,`a3`, `a4` denote the tips of the ancestor tree to which the descendant trees are rooted.

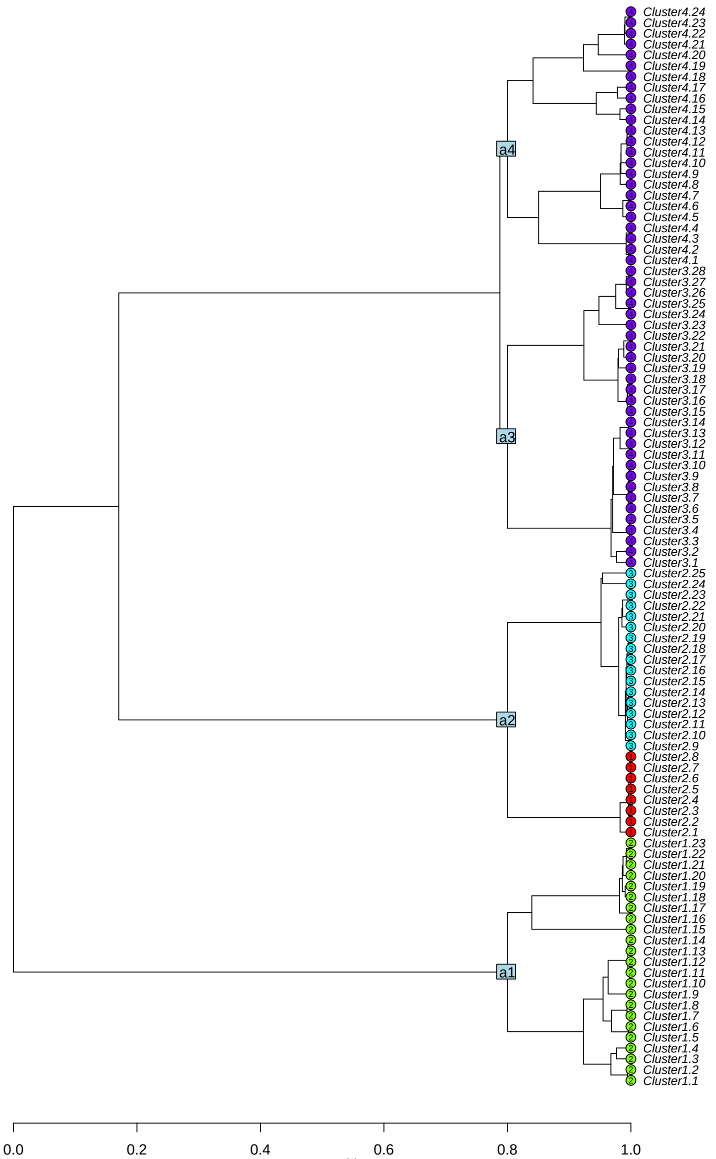

Table S1: Clustering Results for the Gap Procedure on Simulation 1 for varying levels of $r_{\mathbf{AD}}$. The average clustering results (taken over 100 runs) obtained by the Gap Procedure when applied to a four-group simulation with varying values r_{AD} (the ratio of the star-like ancestor tree to descendant tree). The dissimilarity matrix was calculated using the `aK80` distance formula and sequences (of length 800) were mutated according to a GTR + I + Γ model.

$r_{\text{AD}} = \frac{r_{\text{A}}}{r_{\text{A}} + r_{\text{D}}}$	Time (in seconds)	# clusters	# singletons	ARI
0.05	0.1114	21.15	2.69	0.3966
0.10	0.1019	20.51	2.66	0.4156
0.15	0.1027	20.13	2.58	0.4225
0.20	0.1033	19.14	2.35	0.4400
0.25	0.1029	18.60	2.14	0.4517
0.30	0.1033	18.14	2.01	0.4675
0.35	0.1023	18.13	2.09	0.4673
0.40	0.1033	17.34	1.72	0.4843
0.45	0.1021	16.32	1.57	0.5038
0.50	0.1030	15.44	1.40	0.5244
0.55	0.1034	14.55	1.22	0.5471
0.60	0.1032	12.61	0.83	0.5982
0.65	0.1023	10.04	0.64	0.7030
0.70	0.1015	6.97	0.27	0.8408
0.75	0.1017	4.94	0.14	0.9431
0.80	0.1006	4.25	0.04	0.9854
0.85	0.1004	4.01	0.00	0.9997
0.90	0.1028	4.00	0.00	1.0000
0.95	0.1001	4.00	0.00	1.0000

Table S2: Clustering Results for the Gap Procedure on Simulation 1 for varying levels of $r_{\mathbf{AD}}$ while relaxing the star-phylogeny assumption. The average clustering results (taken over 100 runs) obtained by the Gap Procedure when applied to Simulation 1 without forcing a star-phylogeny ancestor tree. The dissimilarity matrix was calculated using the `aK80` distance formula and sequences (of length 800) were mutated according to a GTR + I + Γ model with varying values r_{AD} (the ratio of the ancestor tree to descendant tree).

$r_{\text{AD}} = \frac{r_{\text{A}}}{r_{\text{A}} + r_{\text{D}}}$	Time (in seconds)	# clusters	# singletons	ARI
0.05	0.1225	21.39	2.93	0.3922
0.10	0.1211	20.73	2.66	0.4058
0.15	0.1207	20.25	2.58	0.4132
0.20	0.1207	19.61	2.41	0.4273
0.25	0.1207	19.30	2.41	0.4345
0.30	0.1216	18.85	2.25	0.4464
0.35	0.1228	18.39	2.15	0.4562
0.40	0.1247	17.64	1.82	0.4693
0.45	0.1236	16.81	1.59	0.4840
0.50	0.1220	16.16	1.32	0.4953
0.55	0.1224	15.08	1.16	0.5164
0.60	0.1240	13.69	0.84	0.5416
0.65	0.1231	11.82	0.58	0.5844
0.70	0.1313	9.15	0.46	0.6237
0.75	0.1232	6.84	0.24	0.6661
0.80	0.1229	4.67	0.12	0.6850
0.85	0.1223	3.43	0.01	0.7019
0.90	0.1218	3.16	0.02	0.7224
0.95	0.1213	3.13	0.00	0.7374
