# Supplementary material for: The Gap Procedure: for the identification of phylogenetic clusters in HIV-1 sequence data
Source: BMC Bioinformatics. 2015 Nov 4;16:355. doi: 10.1186/s12859-015-0791-x (PMC4634160; doi:10.1186/s12859-015-0791-x)
Supplement: Additional file 6 — Cluster results for the RAxML and MrBayes clusters on simulated data with T d = 0.4,0.5. Table S1. Cluster results for the RAxML clusters on simulated data with T d = 0.4, 0.5. Table S2. Cluster results for the MrBayes clusters on simulated data with T d = 0.4, 0.5. (PDF 114 kb) [file 12859_2015_791_MOESM6_ESM.pdf]

Cluster results for the RAxML and MrBayes clusters on simulated data with $T_d = 0.4, 0.5$

Table S1: Clustering Results for RAxML on simulated data with $T_d = 0.4, 0.5$. The clustering results (for a single run) obtained by the RAxML when applied to the simulated data. The quoted run times represent the time it takes RAxML to produce a phylogenetic tree. Clade support values are subsequently conducted using 100 bootstrap replicates. RAxML clusters are obtained using a clade support threshold equal to T_c and distance threshold of T_d. The ARI scores in bold indicate which runs performed better than the average score obtained using the Gap Procedure.

Sim	T_c	T_d	Time (in sec)	# clusters	# singletons	ARI
1	90	0.4	2478.966	7	5	0.8606
2	90	0.4	4653.993	9	5	0.8945
3	90	0.4	41584.55	24	6	**0.9764**
4	90	0.4	271593.7	105	33	0.8044
1	90	0.5	2478.966	7	4	0.8757
2	90	0.5	4653.993	9	5	0.8945
3	90	0.5	41584.55	24	6	**0.9764**
4	90	0.5	271593.7	54	2	**0.9922**

Table S2: Clustering Results for MrBayes on simulated data with $T_d = 0.4, 0.5$. The clustering results (for a single run) obtained by MrBayes when applied to the simulated data. The quoted run times represent the time it takes MrBayes to estimate a phylogenetic tree with clade support (i.e., posterior probability) values. MrBayes clusters are obtained using a clade support threshold equal to T_c and a distance threshold of T_d. The ARI scores in bold indicate which runs performed better than the average score obtained using the Gap Procedure.

Sim	T_c	T_d	Time (in sec)	# clusters	# singletons	ARI
1	90	0.4	3324.68	9	2	0.7125
2	90	0.4	4243.64	11	3	0.8529
3	90	0.4	144284.8	38	8	0.8344
4	90	0.4	1328254	122	24	0.6745
1	90	0.5	3324.68	8	2	0.8419
2	90	0.5	4243.64	10	3	0.9011
3	90	0.5	144284.8	24	6	**0.9768**
4	90	0.5	1328254	67	4	0.9448
