# Supplementary material for: The Gap Procedure: for the identification of phylogenetic clusters in HIV-1 sequence data
Source: BMC Bioinformatics. 2015 Nov 4;16:355. doi: 10.1186/s12859-015-0791-x (PMC4634160; doi:10.1186/s12859-015-0791-x)
Supplement: Additional file 7 — Cluster results for the RAxML and MrBayes clusters on the mibc data. Table S1. The threshold values and corresponding clustering results for RAxML on the mibc data. Table S2. The threshold values and corresponding clustering results for MrBayes on the mibc data. (PDF 108 kb) [file 12859_2015_791_MOESM7_ESM.pdf]

Cluster results for the RAxML and MrBayes clusters on the mibc data

Table S1: The threshold values and corresponding clustering results for RAxML on the mibc data. The clustering results obtained by RAxML when applied to the mibc data. The quoted run times represent the time it takes RAxML to estimate a phylogenetic tree. Clusters are obtained using a clade support threshold equal to T_c and a distance threshold of T_d.

T_c	T_d	Time (in sec)	1 ✓	1 ✗	2–4	≥ 5	ARI
90	0.01	56290.46	–	0	75 (175)	6 (409)	0.0176
90	0.02	56290.46	–	0	64 (157)	20 (427)	0.0545
90	0.03	56290.46	–	0	43 (104)	34 (480)	0.1748
90	0.04	56290.46	–	0	28 (69)	44 (515)	0.5421
90	0.05	56290.46	–	0	17 (42)	50 (542)	0.7632
90	0.06	56290.46	–	0	17 (42)	50 (542)	0.7632
90	0.07	56290.46	–	0	12 (30)	51 (554)	0.8977
90	0.08	56290.46	–	0	12 (30)	51 (554)	0.8977
90	0.09	56290.46	–	0	12 (30)	51 (554)	0.8977
90	0.1	56290.46	–	0	12 (30)	51 (554)	0.8977
95	0.01	56290.46	–	0	61 (139)	5 (445)	0.0127
95	0.02	56290.46	–	0	53 (129)	18 (455)	0.0444
95	0.03	56290.46	–	0	37 (89)	31 (495)	0.1230
95	0.04	56290.46	–	0	23 (58)	43 (526)	0.4878
95	0.05	56290.46	–	0	17 (42)	47 (542)	0.6030
95	0.06	56290.46	–	0	17 (42)	47 (542)	0.6030
95	0.07	56290.46	–	0	17 (42)	47 (542)	0.6030
95	0.08	56290.46	–	0	17 (42)	47 (542)	0.6030
95	0.09	56290.46	–	0	17 (42)	47 (542)	0.6030
95	0.1	56290.46	–	0	17 (42)	47 (542)	0.6030
98	0.01	56290.46	–	0	50 (114)	5 (470)	0.0098
98	0.02	56290.46	–	0	49 (117)	13 (467)	0.0271
98	0.03	56290.46	–	0	38 (90)	26 (494)	0.0885
98	0.04	56290.46	–	0	26 (63)	36 (521)	0.2955
98	0.05	56290.46	–	0	20 (49)	40 (535)	0.3720
98	0.06	56290.46	–	0	20 (49)	40 (535)	0.3720
98	0.07	56290.46	–	0	20 (49)	40 (535)	0.3720
98	0.08	56290.46	–	0	20 (49)	40 (535)	0.3720
98	0.09	56290.46	–	0	20 (49)	40 (535)	0.3720
98	0.1	56290.46	–	0	20 (49)	40 (535)	0.3720
99	0.01	56290.46	–	0	43 (100)	5 (484)	0.0081
99	0.02	56290.46	–	0	42 (102)	11 (482)	0.0182
99	0.03	56290.46	–	0	33 (79)	23 (505)	0.0670
99	0.04	56290.46	–	0	24 (61)	31 (523)	0.2014
99	0.05	56290.46	–	0	22 (55)	33 (529)	0.2379
99	0.06	56290.46	–	0	22 (55)	33 (529)	0.2379
99	0.07	56290.46	–	0	22 (55)	33 (529)	0.2379
99	0.08	56290.46	–	0	22 (55)	33 (529)	0.2379
99	0.09	56290.46	–	0	22 (55)	33 (529)	0.2379
99	0.1	56290.46	–	0	22 (55)	33 (529)	0.2379

Table S2: The threshold values and corresponding clustering results for MrBayes on mibc data. The clustering results obtained by MrBayes when applied to the mibc data. The quoted run times represent the time it takes for MrBayes to estimate a phylogenetic tree with posterior probability branch values. Clusters are obtained using a clade support threshold equal to T_c and distance threshold of T_d.

T_c	T_d	Time (in sec)	1 ✓	1 ✗	2–4	≥ 5	ARI
90	0.01	452802.6	–	0	103 (250)	11 (377)	0.0151
90	0.02	452802.6	–	0	86 (217)	33 (410)	0.0698
90	0.03	452802.6	–	0	66 (170)	40 (457)	0.2175
90	0.04	452802.6	–	0	40 (107)	52 (520)	0.4720
90	0.05	452802.6	–	0	8 (22)	59 (605)	0.9011
90	0.06	452802.6	–	0	3 (8)	58 (619)	0.9472
90	0.07	452802.6	–	0	3 (8)	58 (619)	0.9472
90	0.08	452802.6	–	0	3 (8)	58 (619)	0.9472
90	0.09	452802.6	–	0	3 (10)	55 (617)	0.9968
90	0.1	452802.6	–	0	3 (10)	52 (617)	0.9761
95	0.01	452802.6	–	0	94 (225)	11 (402)	0.0130
95	0.02	452802.6	–	0	81 (203)	31 (424)	0.0588
95	0.03	452802.6	–	0	64 (163)	38 (464)	0.1774
95	0.04	452802.6	–	0	43 (116)	49 (511)	0.4114
95	0.05	452802.6	–	0	14 (38)	56 (589)	0.8891
95	0.06	452802.6	–	0	3 (8)	58 (619)	0.9472
95	0.07	452802.6	–	0	3 (8)	58 (619)	0.9472
95	0.08	452802.6	–	0	3 (8)	58 (619)	0.9472
95	0.09	452802.6	–	0	3 (10)	55 (617)	0.9968
95	0.1	452802.6	–	0	3 (10)	53 (617)	0.9864
98	0.01	452802.6	–	0	84 (198)	9 (429)	0.0123
98	0.02	452802.6	–	0	81 (197)	25 (430)	0.0465
98	0.03	452802.6	–	0	63 (153)	35 (474)	0.1454
98	0.04	452802.6	–	0	41 (103)	48 (524)	0.3667
98	0.05	452802.6	–	0	15 (38)	56 (589)	0.8887
98	0.06	452802.6	–	0	3 (8)	58 (619)	0.9472
98	0.07	452802.6	–	0	3 (8)	58 (619)	0.9472
98	0.08	452802.6	–	0	3 (8)	58 (619)	0.9472
98	0.09	452802.6	–	0	3 (10)	55 (617)	0.9968
98	0.1	452802.6	–	0	3 (10)	53 (617)	0.9864
99	0.01	452802.6	–	0	78 (183)	9 (444)	0.0124
99	0.02	452802.6	–	0	77 (184)	25 (443)	0.0441
99	0.03	452802.6	–	0	62 (146)	35 (481)	0.1381
99	0.04	452802.6	–	0	41 (99)	46 (528)	0.3243
99	0.05	452802.6	–	0	14 (33)	55 (594)	0.8765
99	0.06	452802.6	–	0	4 (11)	57 (616)	0.9463
99	0.07	452802.6	–	0	4 (11)	57 (616)	0.9463
99	0.08	452802.6	–	0	4 (11)	57 (616)	0.9463
99	0.09	452802.6	–	0	2 (5)	56 (622)	0.9969
99	0.1	452802.6	–	0	2 (5)	55 (622)	0.9899
